# Quantitative and Qualitative Characteristics of Atherosclerotic Plaques on Carotid Arteries in Patients with Antiphospholipid Syndrome: The Role of MDCT Angiography

**DOI:** 10.3390/diseases11040131

**Published:** 2023-09-28

**Authors:** Jovica Saponjski, Ljudmila Stojanovich, Natasa Stanisavljevic, Aleksandra Djokovic, Radisa Vojinovic, Svetlana Kocic, Simon Nikolic, Predrag Matic, Branka Filipovic, Vuk Djulejic, Vladan Colovic, Nikola Bogosavljevic, Dejan Aleksandric, Dejan Kostic, Biljana Brkic Georgijevski, Miroslav Misovic, Nikola Colic, Dusan Saponjski

**Affiliations:** 1 Faculty of Medicine, University of Belgrade, 11000 Belgrade, Serbiavuk.djulejic@nipro-group.com (V.D.); boga19@gmail.com (N.B.); 2 University Clinical Center of Serbia, 11000 Belgrade, Serbiadrsaska@yahoo.com (A.D.); vladandcolovic@hotmail.co.uk (V.C.);; 3 University Hospital Medical Center “Bezanijska Kosa”, 11080 Belgrade, Serbia; 4 Faculty of Medical Science, University of Kragujevac, 34000 Kragujevac, Serbia; 5 University Clinical Center of Kragujevac, 34000 Kragujevac, Serbia; 6 Clinical Hospital Center Zemun, 11080 Belgrade, Serbia; 7 Faculty of Medicine, University of Kosovksa Mitrovica, 11000 Belgrade, Serbia; simon.nikolic@med.pr.ac.rs; 8 Institute for Cardiovascular Diseases “Dedinje”, 111040 Belgrade, Serbia; 9 University Hospital “Dragisa Misovic”, 11000 Belgrade, Serbia; 10 Institute for Orthopedic Diseases “Banjica”, 11000 Belgrade, Serbia; 11 Military Medical Academy, 11040 Belgrade, Serbia; drdkostic@gmail.com (D.K.); miki_misic@yahoo.com (M.M.); 12 Special Hospital for Cerebrovascular Diseases “Saint Sava”, 11000 Belgrade, Serbia; brkicbiljana15@yahoo.com

**Keywords:** antiphospholipid syndrome, carotid artery, vascular manifestations, MDCTA

## Abstract

Introduction: Antiphospholipid syndrome (APS) is an autoimmune disease characterised by arterious and venous thrombosis, miscarriage, and the presence of antiphospholipid antibodies (aPL) in the blood. As we know, APS is also characterised by accelerated atherosclerotic degeneration with an increased risk of thrombosis in all blood vessels, including the carotid arteries. Carotid artery stenosis can manifest in many different ways. The aim of this study is to present the results of our multidetector computerised tomography angiography (MDCTA) analysis of the carotid arteries in patients with primary and secondary APS compared with a control group. Materials and Methods: This study examined 50 patients with primary antiphospholipid syndrome (PAPS) and 50 patients with secondary antiphospholipid syndrome (SAPS). The results were compared with a control group also comprising 50 patients. The groups were analysed with respect to age, sex and the presence of well-established risk factors for vascular disease. The study was conducted using MDCTA, where we analysed the quantitative and qualitative (morphologic) characteristics of carotid artery lesions. Results: Patients from the control group had significantly elevated levels of cholesterol and triglycerides in comparison with patients with PAPS and SAPS (*p* < 0.001 and *p* < 0.05). The results show that carotid artery lesions were significantly more common in patients with APS (PAPS, *n* = 40, CI95: 0.50–0.75, *p* = 0.0322 and SAFS, *n* = 54, CI95: 0.59–0.80, *p* = 0.0004) than within the control group (*n* = 23). There was a statistically significant difference between patients with APS and the control group with respect to lesions in the distal segments (*n* = 27, CI95: 0.67–0.95, *p* = 0.0001), bulbi and proximal segments (*n* = 21, CI95: 0.84–1.00, *p* = 0.000005). The number of patients with one lesion (L) (*n* = 27) was significantly greater than the number of those with three (*n* = 10, CI95: 0.56–0.86, *p* = 0.0051) or four (*n* = 3, CI95: 0.73–0.98, *p* = 0.00001) lesions. There were also more patients with two lesions (*n* = 24) than those with four (*n* = 3) (CI95: 0.71–0.97, *p* = 0.00005). Carotid artery stenosis was shown as a percentage of the carotid artery lumen diameter (%DS). Stenosis of up to 30%, was more common in patients in the PAPS group (*n* = 12) than in the control group (*n* = 3) (CI95: 0.52–0.96, *p* = 0.0201), while the SAPS group (*n* = 17) had an even larger disparity (CI95: 0.62–0.97, *p* = 0.0017). We observed a highly significant difference in the frequency of stenoses between 30% and 50% DS between the PAPS group (*n* = 24) and the control group (*n* = 7) (CI95: 0.59–0.90, *p* = 0.0023), as well as the SAPS group (*n* = 30) (CI95: 0.65–0.92, *p* = 0.0002). A qualitative analysis of plaque morphology revealed that patients with PAPS had significantly more soft tissue lesions (*n* = 23) compared with calcified lesions (*n* = 2) (CI95: 0.74–0.99, *p* = 0.00003), as well as more mixed plaques (*n* = 9) and calcified plaques (*n* = 2) (CI95: 0.48–0.98, *p* = 0.0348). Patients within the SAPS group had significantly more soft tissue (*n* = 35) than calcified lesions (*n* = 3) (CI95: 0.79–0.98, *p* = 0.00000021), as well as more mixed lesions (*n* = 21) compared with calcified (*n* = 3) (CI95: 0.68–0.97, *p* = 0.0002). Conclusions: Our study shows that subclinical manifestations of carotid artery lesions were more common in patients with APS. We came to the conclusion that MDCTA is an accurate diagnostic method because it is a safe method that provides us with a great quantity of accurate information about the characteristics of atheromatous plaques, which aids us in the further planning of treatment for patients with APS.

## 1. Introduction

Antiphospholipid syndrome (APS) is an autoimmune disease characterised by arterious and venous thrombosis, miscarriage, and the presence of antiphospholipid antibodies (aPL) in the blood. APS can be primary—as a primary disease (PAPS), secondary—in the scope of another autoimmune disease—(SAPS) and catastrophic, which has the worst prognosis of all and a disconcertingly high mortality rate. On the other hand, atherosclerosis is the most common disease in the modern world, and most importantly, manifests as a systemic disease. The course of the disease can be divided into two parts, but as it shows continuous progression, it may be asymptomatic at early stages and clinically evidential at later stages. The first is the subclinical phase, which typically begins in youth and is characterised by a stable, fibrolipid plaque, while the second, clinical phase is characterised by an unstable plaque that can become ulcerated and the site of thrombosis [1,2]. It is well-established that patients with autoimmune diseases have an earlier onset and faster progression of atherosclerosis [3].

The classification criteria for APS (clinical and laboratory) were originally outlined in 1999, in Sapporo, and were then subsequently revised in 2006, in Sydney [4,5,6]. Today, the laboratory criteria used in the diagnosis of APS are: the presence of lupus anticoagulant—a thrombophilic protein in the blood (LAC); anti-cardiolipin antibodies (aCL) of the IgG or IgM class, and aβ2GPI. To meet the diagnosis criteria, these antibodies must be elevated in medium or high titres in two or more separate blood samples, taken at intervals of at least six weeks. The response of the haematopoietic system in these patients is characterised by an increased risk of arterious and venous thrombosis [7,8]. The risk of cardiovascular disease (CVD) in patients with APS is also significantly higher compared to the general population [9].

One of the most common manifestations of APS is the earlier onset atherosclerosis, whose progression leads to the occlusion of blood vessels and can impact any artery or vein. Unsurprisingly, this leads to the gravest consequences when the blood vessels of the head and neck become occluded [9,10]. Because of this, the timely detection of blood vessel lesions is of paramount importance in the management and outcomes of patients with APS. Classic neurologic signs of carotid artery disease are rare at the outset, but the presence of a constellation of otherwise uncharacteristic symptoms caused by cerebrovascular lesions is an indication for further diagnostic exploration. Strokes are the leading cause of disability and mortality in patients with APS [11,12].

Younger patients with APS have a significantly higher risk for CVD compared with the general population, but the risk is altogether far higher in older patients with APS. In addition to the classic risk factors such as diabetes mellitus, arterial hypertension, dyslipidaemia, family history of coronary heart disease, obesity, sedentary lifestyle habits and smoking, APS is a further risk factor for accelerated atherosclerosis. A cerebrovascular event can take many forms, including a transient ischaemic attack (TIA), a reduced visual field, aphasia, and vasculitis up to and including the occlusion of large blood vessels with resulting hemiparesis and hemiplegia [7,9,11,13,14].

For a long time, colour Doppler ultrasonography (DUS) was the only non-invasive imaging method used to diagnose stenotic lesions in peripheral blood vessels. DUS has been shown to be a reliable modality for the examination of the larger blood vessels of the extremities, as well as carotid arteries. The modern approach to the detection of blood vessel pathology makes use of multidetector computed tomography angiography (MDCTA). The consensus today is that a 64 CT scanner is the imaging modality of choice, since it is excellent for the visualisation of smaller and larger blood vessels, their anatomic characteristics, including vitally important virtual angiography, and the morphology of atheromatous plaques.

The aim of this study is to present the results of our MDCTA quantitative and qualitative analysis of the carotid arteries in patients with primary and secondary APS compared with a control group.

## 2. Materials and Methods

This case–control study comprised 50 patients (pts) with PAPS (39 women, 21 men), 50 patients with SAPS (31 women, 19 men) and 50 patients as a control group (14 women, 36 men), in which all participants were diagnosed with atherosclerotic plaques, but confirmed to have no autoimmune diseases. We discovered that there was no significant difference in age between the groups of patients with APS and the patients in the control group (*p* < 0.05), while the difference with respect to sex was significant (*p* < 0.05). After clinical examination, the main indication for MDCTA in patients with APS was the evaluation of carotid artery lesions because of neurologic symptoms. The control group comprised patients without APS for whom an MDCTA was indicated because of peripheral vascular disease. The criteria for exclusion were pregnancy or lactation, severe respiratory, heart or kidney failure, and a severe infection or fever.

APS was diagnosed according to the Sydney Classification Criteria set forth in 2006. In conjunction with clinical signs and symptoms, laboratory testing has established serum and plasma reference and cut-off values for phospholipid-dependent coagulation tests that enable the detection of lupus anticoagulant (LAC), and immunofluorescence tests (ELISA) for anti-cardiolipin antibodies (aCL) of the IgG and IgM classes, as well as anti-ß2 glycoprotein-I of the IgG and/or IgM class.

A standard laboratory analysis and assessment of the atherosclerosis risk factors (dyslipidaemia, hyperglycaemia, smoking, arterial hypertension and family history) was conducted for all study participants. After clinical examination, DUS and MDCTA were used to examine the carotid arteries of all participants.

MDCTA examination was conducted bilaterally for the common carotid artery segments (proximal segment, distal segment and bulbus), as well as both the internal carotid artery and external carotid artery. We used whole-body angiography (WBA) for a period of 26 s, i.e., less than a minute, with the application of 70 to 120 mL of contrast agent (Ioversol 350), followed by 30 mL of physiological solution. The results were analysed by an integrated system of quantitative peripheral angiography, giving us the stenosis diameter. The changes in blood vessels analysed in patients with subclinical disease were unknown before our examination (protocol taken from the Congress of Radiologists in Chicago–RSNA, 2004).

All lesions that we detected in the scope of our carotid MDCTA scans were classified into four groups based on the percentage of decrease in the artery lumen diameter (%DS): (1) 0–30%, (2) 31–50%, (3) 51–70%, (4) >70%. Moreover, we analysed the plaque morphology visualised within each scan and divided all plaques into three discrete groups: soft tissue lesions, calcified lesions and mixed type lesions.

We also categorised our patients into four discrete groups based on the number of lesions found: (1) pts with one lesion, (2) pts with two lesions, (3) pts with three lesions, (4) pts with four lesions at different segments (proximal common carotid artery, distal common carotid artery, carotid artery bulbus, internal carotid artery and external carotid artery). The results were compared quantitatively and qualitatively within each group, as well as between different groups.

Descriptive statistical analysis was used for the basic statistical analysis of our populations’ characteristics. Further analysis was carried out depending on whether the data were normally distributed or not. We tested for normality using the Kolmogorov–Smirnov test. If the data were shown to have a normal distribution, we used parametric analysis of variance (one-way analysis of variances) for comparing differences between the experimental groups. In the event that the data were not normally distributed, we used a nonparametric test of variance (Kruskal–Wallis analysis of variance). If there were statistically significant differences between groups, they were compared with each other by the parametric Tukey test, or the non-parametric Dunn’s Multiple Comparison Test or chi-square test. Statistical analysis was performed using the statistical package GraphPad Prism, version 6.00 per Windows (GraphPad Software, San Diego, CA, USA; https://www.graphpad.com/, accessed on 23 September 2023).

## 3. Results

With respect to the traditional risk factors for atherosclerosis, patients from the control group had significantly elevated levels of cholesterol in comparison with patients with PAPS and SAPS (*p* < 0.001). Also of note, patients from the control group had significantly elevated levels of triglycerides in comparison with patients with PAPS and SAPS (*p* < 0.05). With respect to the other risk factors that we analysed, no other significant differences were found. This case–control study comprised 50 patients (pts) with PAPS (mean age: 53 ± 11 years; 39 women, 21 men), 50 patients with SAPS (mean age: 53 ± 9 years; 31 women, 19 men) and 50 patients as a control group (mean age: 54 ± 8 years; 14 women, 36 men) (Table 1).

Patients with APS were shown, using MDCTA, to have significantly more atherosclerotic carotid lesions (L) than patients in the control group. Patients with PAPS, as well as patients with SAPS, had significantly more atherosclerotic lesions compared to patients in the control group. Our results show that significantly more patients in the PAPS group had atherosclerotic lesions than patients in the control group (40 L vs. 23 L, *p* <0.05), but also that patients in the SAPS group had more lesions than patients in the control group (54 L vs. 23 L, *p* < 0.001).

The MDCTA analysis showed that among the 150 patients enrolled in this study, there were 117 lesions (40 in the PAPS group, 54 in the SAPS group and 23 in the control group) with varying degrees of carotid artery stenosis. A statistically significant difference between groups was observed in the proximal segments of both carotid arteries. The frequency of proximal left common carotid artery (LCCA) lesions and proximal right common carotid artery lesions was significantly higher in the PAPS group (5/50 vs. 0/50 patients, *p* < 0.05) as well as the SAPS group (6/50 vs. 0/50 patients, *p* < 0.05) when compared to the control group. A similar difference was also observed when comparing the distal segments and bulbi of both common carotid arteries (Table 2).

After examining the numbers of carotid artery lesions, we determined that our sample contained more patients without any lesions than patients with one or more carotid artery lesions of varying degrees of stenosis (86/150 vs. 64/150, *p* < 0.05). We found that the control group contained significantly fewer patients with carotid artery lesions than patients without (16/50 vs. 34/50 patients, *p* < 0.01), which is exceedingly statistically significant (Table 3).

While examining the distribution of the number of lesions across all three groups (PAPS, SAPS, control), we observed an interesting trend. The number patients with a single lesion were significantly higher than the number of patients with three (27 pts vs. 10 pts, *p* < 0.01) and four lesions (27 pts vs. 3 pts, *p* < 0.001). It was also shown that there were more patients with two lesions than those with four (24 vs. 3 patients, *p* < 0.001). The number of patients with two lesions was higher than the number of patients with three lesions (24 pts vs. 10 pts, *p* < 0.05). We did not find any statistically significant differences between our groups for patients with one lesion and two lesions.

After considering the number of lesions within each group (PAPS, SAPS, control), we found that the PAPS group contained more patients with one lesion than patients with three (7 pts vs. 2 pts, *p* < 0.01), as well as more patients with two lesions than with four (13 pts vs. 0 pts, *p* < 0.001) and with three (13 pts vs. 2 pts, *p* < 0.01). The SAPS group contained significantly more patients with one lesion than with four (10 vs. 3 patients, *p* < 0.01). The control group also contained significantly more patients with one lesion than with three or four (10 vs. 1 vs. 0 patients, *p* < 0.01), as well as more of those with two lesions than patients with four lesions (5 vs. 0 patients, *p* < 0.05) (Table 4).

In our analysis of the degree of carotid artery stenosis, shown as a percentage decrease in the lumen diameter (%DS), we demonstrated a very high statistical significance with respect to the number of patients in the SAPS group who had carotid artery stenosis in comparison to the control group (54 vs. 23 lesions, *p* < 0.001). The significance was somewhat less pronounced in the case of the PAPS group (40 vs. 23 lesions, *p* < 0.05). When we analysed the results in the PAPS group for patients with a 0–30% DS, we noticed that there were significantly more patients with this degree of stenosis than in the control group (12 vs. 3 lesions, *p* < 0.05). Meanwhile the SAPS group had an even larger disparity (17 vs. 3 lesions, *p* < 0.01). A significant difference was inferred between the PAPS group and the control group for 31 to 50% DS (24 vs. 7 lesions, *p* < 0.01), as well as between the SAPS group and the control group (31 vs. 7 lesions, *p* < 0.001). No significant difference was found with respect to 51 to 70% DS between groups, nor was any found for >71% DS (Table 5).

All lesions, based on their plaque micromorphology, were qualitatively categorised into three discrete groups: soft tissue plaques with minimal calcification, mixed type plaques in which calcium and other cellular and extracellular elements were in roughly equal quantities, and calcified plaques, which were composed mainly of calcium deposits. Of the 117 lesions that we analysed, 62 (52.9%) lesions were of the soft tissue type, 39 were mixed (33.3%) and 16 were calcified (13.8%). The PAPS group of patients was found to have significantly more soft tissue lesions than mixed lesions (23/34 vs. 9/34, *p* < 0.01) and calcified lesions (23/34 vs. 2/34, *p* < 0.001). There was also a statistically significant difference in the frequency of mixed plaques versus calcified plaques (9/34 vs. 2/34, *p* < 0.05). The SAPS group, in total, contained 59 plaques of varying structure. The SAPS group of patients was found to have significantly more soft tissue lesions than calcified (35/59 vs. 3/59, *p* < 0.001). There was also a statistically significant difference in the frequency of mixed plaques versus calcified plaques (21/59 vs. 3/59, *p* < 0.001). The control group, in total, contained 24 plaques of varying structure (Table 6).

The control group contained 11 calcified plaques of the 24 that were observed in total. This was significantly more common than in the PAPS group (11 vs. 2, *p* < 0.01) and the SAPS group (11 vs. 3, *p* < 0.05).

## 4. Discussion

Atherosclerosis is the leading problem in modern medicine, and to this day, we are in search for the magic bullet that will lower its unacceptably high prevalence. Unfortunately, it is well known that patients with autoimmune diseases can have an increased risk of vascular disease, which can lead to increased mortality. Novel imaging methods are necessary for early detection, assessment and management in patients with these diseases. In our study, which comprised 100 patients with APS (50 with PAPS, 50 with SAPS), we analysed the frequency as well as the degree of carotid artery stenosis and the morphologic characteristics of the plaques in question, discovered with the aid of MDCTA. It must be emphasised that our sincere wish was to demonstrate the importance of early detection in the case of carotid artery disease and above all, to elucidate the microstructure of the culprit plaque. Research and experience have long since established the vulnerability of soft tissue plaques, as well as even mixed-type plaques, to be far greater than that of calcified plaques—even those whose %DS is lower than 70%. It was of particular interest to us to explore the role of minimally invasive methods in the early detection of subclinical manifestations in patients with APS, as well as their continued role in the surveillance of this disease’s progression. In the analysis of our results and known risk factors for CVD, we observed that some risk factors were significantly greater in the control group. This implies that the classic risk factors for CVD are of no significant importance in the accelerated progression of atherosclerosis in patients with APS. Of course, every instance of increased risk factors in patients with APS must be managed, and biochemical markers of disease must be brought within the reference range.

In a previous, wide-ranging study, Schoenfeld et al. analysed various cohort studies and concluded that epidemiologic studies so far have implied a link between the classic risk factors for CVD (hyperlipidaemia, cigarette smoking, advanced age, arterial hypertension, male sex, and elevated C-reactive protein) and the increased risk for CVD in patients with systemic lupus erythematosus (SLE). The authors further noted that previous studies have not examined all these risk factors simultaneously within the same population, so a calculation of their relative risk is not possible. In addition to traditional CVD risk factors, several SLE-associated factors have also been predictive of CVD risk in past cohort studies [10,14].

MDCTA grants us access to a wealth of highly precise information that is unavailable when using other diagnostic tools [15,16]. From our results, we can conclude that the frequency of blood vessel lesions is much greater in patients with APS than in patients within the control group. MDCTA is the agreed-upon gold standard for examining the progression of APS as well as the early detection of blood vessel lesions [16,17]. Our results have shown that carotid artery lesions are statistically more common in patients with APS. The majority of lesions in patients with APS lead to stenoses of below 50% of the carotid artery lumen, and these lesions are predominantly of the soft tissue and mixed types.

Across all groups, the greatest statistical differences in carotid artery lesions were found in the distal segments and bulbi of the common carotid arteries, but also in the internal carotid artery. This can be explained by their specific anatomy, curvature and length. Past research and experience therefore illustrate the importance of the prompt diagnosis and treatment of carotid artery stenosis, the consequences of which may be lethal. It has been shown that neurologic symptoms in patients with APS can range from non-existent to severe, and that their outcomes can be fatal [18,19,20,21].

At a glance, our study contained a relatively meagre number of APS patients with carotid artery lesions whose %DS was above 50% (only 10 out of 100 patients). This result was not statistically significant, so it was not described in our results. The control group contained 13 predominantly calcified lesions with a %DS greater than 50% (6/13) or 70% (7/13). While these values may seem of lesser importance, we must emphasise that the early detection of carotid artery stenosis increases the probability of successful management [21,22,23].

By comparing our results with previous findings, we have shown that MDCTA is a superior method compared to DUS, which cannot be used to visualise smaller blood vessels and cannot provide the precise quantitative and qualitative characteristics of the atheromatous plaque under examination [23,24,25,26]. Invasive angiography was, despite its possible complications during and after the procedure, until recently, the gold standard. At this moment in time, its role is that of one of various interventional radiology procedures available. When we compare the two methods, MDCTA is the superior method, mainly due to its non-invasive nature and minimal risk [27,28,29]. In the scope of this study, all participants were examined using a DUS, but the results of this were unsatisfactory and did not carry the same statistical relevance, particularly in the proximal segments of the common carotid arteries, where nearby bone tissue can obscure the view, as well as with highly tortuous arteries.

Magnetic resonance angiography (MRA) is not routinely used in diagnostics, but we hope that it may find itself in wider use in future as a non-invasive, alternative method that spares patients the irradiation that comes with imaging methods that rely on the use of x-ray radiation. The realisation of MRA’s full clinical potential requires the continuation of study efforts and further improvements by the way of increased technique efficiency, and better spatial and contrast resolution and artefact suppression [30,31,32,33].

## 5. Conclusions

MDCTA is a modern, minimally invasive imaging method with practically no complications (except for the possibility of an allergic reaction to contrast material); therefore, it is considered a low-risk diagnostic tool. MDCTA, unlike DUS, eliminates some errors of interpretation and allows us to visualise the precise quantitative and qualitative characteristics of the atheromatous plaque under examination, which is of particular importance for the assessment of disease progression and management in patients with APS. Surveillance of de novo lesions, whose symptoms are often discrete and easy to miss, can prevent the unwanted outcome of arterial occlusion when promptly treated with interventional radiology procedures, most often stenting.

## Figures and Tables

**Table 1:** Baseline study population characteristics.

Risk Factors	PAPS	SAPS	Controls	*p*
Age (years)	52.98 ± 11.24	53.26 ± 9.29	54.47 ± 8.04	*p* ≥ 0.05
Cholesterol levels (mmol/L)	2.86 ± 1.04	2.85 ± 0.86	7.94 ± 0.57	*p* < 0.001
Trygliceride levels (mmol/L)	1.33 ± 0.49	1.36 ± 0.48	3.71 ± 0.56	*p* < 0.05
Glycaemia (mmol/L)	5.05 ± 1.04	4.92 ± 0.69	5.09 ± 0.85	*p* ≥ 0.05

**Table 2:** Number of arterial stenoses in the study population (*n* = 117 lesions).

	PAPS (50 Patients)	SAPS (50 Patients)	Control (50 Patients)
DACC prox.	5 ^A^	6	0 ^A^
LACC prox.	5 ^A^	5	0 ^A^
DACC dist. and bulbus	5 ^A^	8 ^A^	2 ^AA^
LACC dist. and bulbus	8 ^A^	6 ^A^	3 ^AA^
DACI	6	11	6
LACI	7	8	9
DACE	3	5	2
LACE	2	5	1
Total (*n* = 117)	40 lesions ^A^	54 lesions ^y^	23 lesions ^Ay^

DACC prox. (proximal segment of the right common carotid artery), LACC prox. (proximal segment of the left common carotid artery), DACC dist. and bulbus (distal segment and bulbus of the right common carotid artery), LACC dist. and bulbus (distal segment and bulbus of the left common carotid artery), DACE (right external carotid artery), LACE (left external carotid artery). A = *p* < 0.05, **y** = *p* < 0.001.

**Table 3:** Frequency of carotid artery lesions.

	Patients with Lesions	Patients without Lesions
PAPS (40 lesions, 50 patients)	22	28
SAPS (54 lesions, 50 patients)	26	24
Control (23 lesions, 50 patients)	16 ^a^	34 ^a^
Total	64 ^A^	86 ^A^

A = *p* < 0.05; **a** = *p* < 0.01.

**Table 4:** Frequency of different numbers of lesions by group.

	Patients with 1 Lesion	Patients with 2 Lesions	Patients with 3 Lesions	Patients with 4 Lesions
PAPS 40 lesions, 22/50 patients	7 ^a^	13 ^yc^	2 ^c^	0 ^ay^
SAPS 54 lesions, 50 patients	10 ^a^	6	7	2 ^a^
Control 23 lesions, 50 patients	10 ^a^	5 ^A^	1 ^a^	0 ^aA^
Total	27 ^yA^	24 ^YA^	10 ^aA^	3 ^yY^

A = *p* < 0.05; a, c = *p* < 0.01; y, Y = *p* < 0.001.

**Table 5:** Frequency of lesions by %DS in each group.

DS%	PAPS	SAPS	Control
0–30%	12 ^A^	17 ^a^	3 ^Aa^
30–50%	24 ^a^	30 ^y^	7 ^ay^
50–70%	3	3	7
>70%	1	3	6
Total (*n* = 117 lesions)	40 ^A^	54 ^y^	23 ^yA^

A = *p* < 0.05; a = *p* < 0.01; **y** = *p* < 0.001.

**Table 6:** Lesion morphology by group.

Group	Calcified	Mixed	Soft Tissue	*n*
PAPS	2 ^Ax^	9 ^Aa^	23 ^xa^	34
SAPS	3 ^xy^	21 ^x^	35 ^y^	59
Control	11	9	4	24
*n* = 117	16	39	62	117

Statistical significance is shown in the same letters per row. A = *p* < 0.05; a = *p* < 0.01; x, y = *p* < 0.001.

## Data Availability

Data sharing is not applicable.
